# Catalytic Strategies for the Cycloaddition of CO_2_ to Epoxides in Aqueous Media to Enhance the Activity and Recyclability of Molecular Organocatalysts

**DOI:** 10.3390/molecules29102307

**Published:** 2024-05-14

**Authors:** Niracha Tangyen, Wuttichai Natongchai, Valerio D’Elia

**Affiliations:** VISTEC Advanced Laboratory for Environment-Related Inorganic and Organic Syntheses, Department of Materials Science and Engineering, School of Molecular Science and Engineering, Vidyasirimedhi Institute of Science and Technology (VISTEC), Rayong 21210, Wangchan, Thailand; niracha.t_s20@vistec.ac.th (N.T.); wuttichai.n_s16@vistec.ac.th (W.N.)

**Keywords:** cycloaddition reaction, cyclic carbonates, CO_2_ conversion, catalysis, aqueous media, “on water” reactions

## Abstract

The cycloaddition of CO_2_ to epoxides to afford versatile and useful cyclic carbonate compounds is a highly investigated method for the nonreductive upcycling of CO_2_. One of the main focuses of the current research in this area is the discovery of readily available, sustainable, and inexpensive catalysts, and of catalytic methodologies that allow their seamless solvent-free recycling. Water, often regarded as an undesirable pollutant in the cycloaddition process, is progressively emerging as a helpful reaction component. On the one hand, it serves as an inexpensive hydrogen bond donor (HBD) to enhance the performance of ionic compounds; on the other hand, aqueous media allow the development of diverse catalytic protocols that can boost catalytic performance or ease the recycling of molecular catalysts. An overview of the advances in the use of aqueous and biphasic aqueous systems for the cycloaddition of CO_2_ to epoxides is provided in this work along with recommendations for possible future developments.

## 1. Introduction

Despite the availability of a large variety of catalytic reactions for the conversion of CO_2_ to chemicals [1,2,3,4], the cycloaddition reaction of CO_2_ to epoxides has remarkably surged in the last two decades to become the most intensively investigated nonreductive CO_2_ conversion process [5,6,7,8,9,10], and one of the most universally studied catalytic processed involving CO_2_ in absolute terms [11]. This is due to several factors, such as the fact that it can be carried out under reasonably mild reaction conditions (most usually 25–120 °C, 1–30 bar CO_2_ pressure) and even under ambient conditions [12,13,14,15,16,17], it uses readily available epoxide substrates [18], and, unlike most reductive CO_2_ conversion processes [19,20,21,22], it does not require the use of flow reactors and/or H_2_ as a hazardous reducing agent [23]. More importantly, the produced cyclic organic carbonates are emerging as valuable compounds for several crucial applications. Cyclic carbonates such as ethylene carbonate, propylene carbonate, and fluorinated cyclic carbonates are essential components of lithium batteries [24,25,26]. Cyclic carbonates serve as intermediates for the synthesis of important polymers through new routes that are less toxic than their traditional counterparts. Indeed, ethylene carbonate is produced in the Asahi–Kasei process as a precursor of linear carbonates during the phosgene-free synthesis of polycarbonates [27,28]. A variety of compounds containing multiple cyclic carbonate groups has been investigated for the synthesis of polyhydroxy urethanes (PHUs) by step-growth polymerization with polyamines leading to increasingly viable isocyanate-free routes towards traditional polyurethane replacements [29,30,31,32,33]. Other important applications of cyclic organic carbonates are as solvents [34,35], surfactants [36], demulsifiers [37], and precursors of more complex molecules [38,39]. Moreover, the fact that the catalytic cycloaddition of CO_2_ to epoxides can be carried out by using diluted or even flue gas CO_2_ [40,41] has led to the suggestion that cyclic carbonate synthesis could serve as a way to convert waste CO_2_ into useful compounds, averting its atmospheric release [42,43]. While such a prospect is challenged by the oil-based nature of most industrially available epoxides [44] and market limitations of the carbonate products [45], it is clear that the widespread adoption of cyclic carbonates as multipotent compounds and of their synthesis as a carbon sink, require highly available, inexpensive, and sustainable catalytic strategies [46,47].

**Figure 1:**
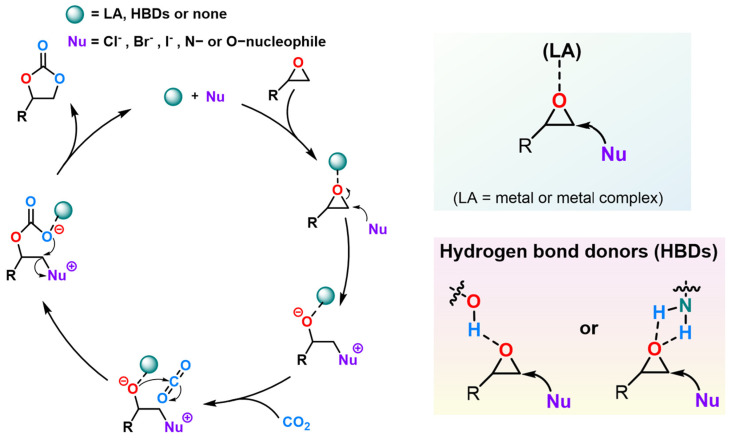
General mechanism of the cycloaddition reaction of CO_2_ to epoxides catalyzed by nucleophilic catalysts in the absence or in the presence of activators (**Left**). Examples of catalytic components that are generally used for the activation of epoxides, i.e., Lewis acidic metals (LA) or HBDs (**Right**).

While different catalytic strategies exist [48,49,50], the cycloaddition of CO_2_ to epoxides under mild conditions is typically catalyzed by nucleophiles such as halide (Cl, Br, and I) anions [51,52], or nucleophilic pyridines [53,54,55], and other *N*-[56] or O-nucleophiles [57] whose function is to ring-open the epoxide to generate an alkoxide for the subsequent step of CO_2_ activation in the form of a hemicarbonate that is formed upon a nucleophilic attack by the produced alkoxide (Figure 1, Left) [58]. Along with the nucleophile, the catalytic cycloaddition reaction is strongly accelerated by the presence of additional catalytic components serving as metal-based Lewis acids [40,59,60,61] or as H-bond donors [62,63,64,65] (HBDs) for the activation of the epoxide (Figure 1, Right) leading to a reduction in the ring-opening barrier and allowing the reaction to proceed under milder conditions [58,66]. As an effect, most catalytic systems for the target cycloaddition reaction are binary combinations of nucleophiles and Lewis acidic complexes [43,67] or nucleophiles and HBDs [8,68]. The dual catalytic moieties may be placed in two different molecules or as distinct groups of the same multifunctional compound leading to single-component catalysts [69,70,71,72].

In particular, organocatalytic HBDs, while typically not as active as metal-based Lewis acids in the epoxide activation step [43,73], are a convenient choice for the cycloaddition of CO_2_ to epoxides because, as organocatalytic metal-free compounds, they are generally highly stable under ambient conditions and do not pose a risk of product contamination by metal leaching [74]. Moreover, HBDs are often simple organic molecules [63,64,75,76], that, unlike most metal organic complexes and frameworks [77,78], do not require the synthesis of sophisticated ligand systems. Readily available HBD materials [79,80,81], and even biobased HBDs [46,82,83], have been reported for the synthesis of cyclic carbonates under atmospheric pressure in the presence of a very small amount (≤0.5 mol%) of homogeneous nucleophilic additives. Organocatalysts are also typically moisture-resilient as they do not undergo the typical water-induced degradation processes of metal-based compounds [84].

Interestingly, the most simple, ubiquitously available, and inexpensive HBD is water itself, a fact which has led to its increasing involvement in numerous catalytic processes either as a medium or as a beneficial additive [85,86,87,88,89]. However, the role of water in chemical reactions extends far beyond that of a mere HBD; due to its high polarity and general lack of miscibility with most organic substrates, the presence of water in organic reactions leads to the confinement of nonpolar substrates into hydrophobic domains with strong effects on the outcome of the catalytic process in terms of rate enhancement and selectivity [90,91,92,93]. This aqueous chemistry traces back to the original discovery of Rideout and Breslow [94] on the hydrophobic acceleration of the Diels–Alder reaction that was subsequently developed by Sharpless et al. [95], the latter referring to “on water” reactions as opposed to reactions carried out in water using water-soluble substrates. The development of “on water” chemistry sparked a gold rush to investigate the “on water” version of various catalytic reactions [92,96,97,98]. Such a trend closely paralleled the rise of the field of organocatalysis (due to the fact that most “on water“ studies used water-tolerant, metal-free organocatalysts) [99,100,101]. In these works, the acceleration of organic reactions in aqueous media was attributed to the combination of the hydrogen-bonding co-ordination and activation of the substrates by water [92,102,103], and to the hydrophobic confinement of the reagents leading to “more compact” transition states [90,98,104,105].

Despite such crucial developments, until recently, very limited studies have targeted the enhancement in the cycloaddition of CO_2_ to epoxides by the addition of water or by the use of aqueous systems. This reluctance towards the inclusion of water in the latter cycloaddition process is likely due to the fact that the earlier catalytic systems for the cycloaddition process were based on notoriously water-sensitive Lewis acidic metal complexes [60,106,107]. Therefore, in this work, we briefly review the emerging approaches in carrying out the target catalytic cycloaddition process in the presence of water as an HBD catalytic component, as an additive or as a medium according to the amount used relative to the substrate. We show that, for specific catalytic systems, the use of aqueous systems is a way to increase the catalytic activity, catalyst recoverability, or both, making water a useful tool that can serve, in the future, in pushing the boundaries of the catalytic CO_2_ conversion to cyclic carbonates and, possibly, to other substrates.

## 2. Results

### 2.1. Water or No Water?

Firstly, it is worth considering whether water should be included in the cycloaddition of CO_2_ to epoxides at all. The latter reaction is a classic example of an atom-economic sustainable solventless reaction where the substrate is also the reaction medium that is progressively replaced by the product as a more polar medium [108]. Given the polar nature of the starting material, and the high polarity of the carbonate product, by far the majority of the molecular catalysts is soluble in the reaction medium (possibly due to the complexation by several molecules of epoxide), or it gets dissolved by increasing the reaction temperature and/or upon the formation of reaction intermediates [40], or of small amounts of carbonate product. Compared to the solventless cycloaddition reactions, the use of solvents, for instance, to solubilize one of the catalytic components, is obviously less sustainable as it affects the atom economy of the overall process and generates potentially toxic solvent wastes, especially when solvents such as acetonitrile or DMF are added [109]. Similarly, the use of water as a mere additive just to adjust the solubility of the catalyst is clearly not advantageous or sustainable compared to solventless reactions as it would generate contaminated water phases requiring the removal of the organic pollutants prior to disposal [110]. Therefore, the addition of water in the reaction medium is desirable only if it significantly accelerates the reaction or if it is beneficial under other aspects such as by inducing hydrophobic effects as in the “on water” reactions, or by facilitating the recovery of the catalyst as discussed in the following sections.

**Scheme 1:**
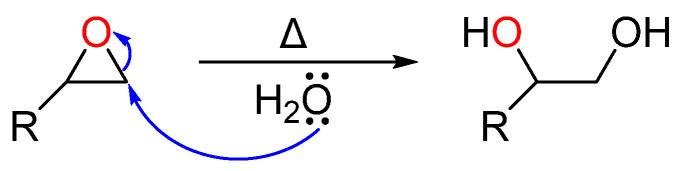
Epoxide hydrolysis generating diols compound.

Another aspect is whether the presence of water interferes with the catalytic activity; mixed literature results are available over the effect of the addition of small amounts of water to the cycloaddition of CO_2_ to epoxides by various catalytic systems. For instance, Yang et al. showed that, when using a catalytic system consisting of the μ-oxo- tetranuclear zinc cluster Zn_4_(OCOCF_3_)_6_O/TBAI (TBAI: tetrabutylammonium iodide) under ambient conditions, the addition of water of up to 3 mol% was well-tolerated but the further addition of just 1 mol% water led to a strong decrease of catalytic performance from over 80% to about 50% [111]. Nevertheless, the addition of up to 10 mol% water to the organocatalytic cycloaddition reaction catalyzed by ascorbic acid/TBAI under ambient conditions had no significant effects, with the epoxide conversion remaining close to 70% for up to 8 mol% water and slightly dropping to 65% with 10 mol% water [63]. In the case of the cycloaddition of CO_2_ to epoxides catalyzed by group I, II halide salts and nucleophilic pyridines at 60 °C, 1 bar CO_2_, the addition of water of up to 10 mol% had a slightly beneficial effect on epoxide conversion, increasing from about 60 to 80%, but with a small reduction in carbonate selectivity (from 93 to 88%) due to the formation of diols (from epoxide hydrolysis) [55]. Indeed, treatment in hot water (T > 60 °C) is known to lead to the opening of epoxides in the form of diols (Scheme 1) [112]. Based on these data, the presence of water in the cycloaddition reaction over a certain concentration threshold may be detrimental to catalytic systems susceptible to hydrolysis while it is less likely to impact water-stable systems such as organocatalysts and ionic compounds. At the same time, the occurrence of significant hydrolytic processes leading to undesired by-products should be minimized. 

### 2.2. Catalytic Effect of Sub-Stoichiometric Amounts of Water

In this section, the reports on the acceleration of the cycloaddition of CO_2_ to epoxides by sub-stoichiometric amounts of added water (up to 33 mol%) are discussed. It is worth noting that, unless using distilled epoxides, which is rarely carried out, the substrate of the cycloaddition process always contains trace amounts of water. However, the water content of fresh epoxides is generally in the 20–50 ppm range corresponding to << 1 mol% H_2_O. 

To the best of our knowledge, the first report on the beneficial effect of water on the cycloaddition of CO_2_ to epoxides by molecular catalysts was reported by Sun et al. [113]. The authors showed that the addition of 33 mol% water to the cycloaddition reaction of CO_2_ to propylene oxide (PO) catalyzed by a variety of ionic compounds (sodium halide salts, tetrabutylammonium halide salts, 1-butyl-3-methyl-imidazolium halides, and butyltriphenylphosphonium halides) under relatively harsh conditions (125 °C, 20 bar) led, in nearly all cases, to a remarkable increase not only in PO conversion but also in propylene carbonate (PC) selectivity compared to the water-free reactions. This result indicates that very high carbonate selectivities can be obtained in the presence of water even at very high temperatures, possibly because the hydrolysis of the epoxide is strongly inhibited by the dilution of water in the organic medium. The increase in catalytic activity was particularly remarkable for iodide salts that displayed a stronger enhancement in performance compared to the other halide ions. The reaction catalyzed by PPh_3_BuI (butyltriphenylphosphonium iodide) and water led to four times the PO conversion in 1 h as the reaction carried out in the absence of water (see Table 1, entries 1–4 for selected results). According to kinetic measurements, the aqueous reaction was about seven times faster than the corresponding reaction without added water. The authors also showed that the formation of diols occurred by increasing the amount of water above the optimized threshold of 33 mol%, leading to a drop in carbonate selectivity and yields. The author proposed a role of water as an HBD to activate the epoxide for the ring-opening step of the reaction mechanism. Substantially similar results were reported in a subsequent paper by Zhou et al. [114], showing that the performance of simple halides such as NaI and LiI in the synthesis of propylene carbonate under harsh conditions (120 °C, 20 bar) was strongly enhanced by replacing the anhydrous alkali salts with their hydrated counterparts (NaI·2H_2_O; LiI·2H_2_O; see Table 1, entries 5, 6 for the case of NaI and NaI·2H_2_O). Through DFT calculations, Wang et al. showed that the energy barrier for the rate-determining step of the epoxide ring-opening in the cycloaddition reaction of CO_2_ to ethylene oxide catalyzed by TBAB (tetrabutylammonium bromide) decreased by about 7.5 kcal/mol in the presence of water due to its role as an HBD [115]. 

In a different work, Mazo and Rios [119] applied the methodology developed by Sun et al. [113] for the challenging carbonation of epoxidized soybean oil (ESBO) [120,121,122]. The cyclic carbonate product (CSBO) is a crucially important precursor for the synthesis of non-isocyanate polyurethanes in the form of PHUs [29,30,32]. The addition of 33 mol% water relative to the epoxide accelerated the cycloaddition reaction of CO_2_ to ESBO at 120 °C catalyzed by TBAB under a continuous CO_2_ flow leading to a reduction in the *t*_1/2_ (time for the conversion of 50% epoxides in ESBO to cyclic carbonate moieties) of 70%.

In past works, the role of water in enhancing the catalytic performance of quaternary ammonium halide salts had been generally investigated under harsh reaction conditions. More recently, Alassmy and Pescarmona revisited the role of water in the cycloaddition of CO_2_ to epoxides under mild conditions (45 °C, 10 bar) [116] but in a longer reaction time (18 h), and with lower conversion values, compared to the previous study by Sun et al. (Table 1, entries 1, 2) due to the stark decrease in temperature impacting the overall reaction kinetics. While, under such conditions, organic halides such as TBAI and PPNI (bis(triphenylphosphine)iminium iodide) displayed negligible catalytic activity, the addition of water led to moderate and selective substrate conversion (Table 1, entries 7, 8). Within the latter study, the authors showed that, under identical conditions, the addition of 14 mol% water had a comparable effect as the presence of much lower loadings (0.25 mol%) of more active state-of-the-art HBDs such as ascorbic acid [63] and phenol derivatives [14,76]. Importantly, and at variance with previous reports, the enhancement in catalytic performance by water under mild conditions was observed only in the case of iodide compounds, whereas the presence of water had detrimental effects on substrate conversion when using bromide and chloride compounds. This observation was attributed to the I^−^ > Br^−^ >Cl^−^ order of the nucleophilicity of the halide anions in water due to shielding of the smaller anions by the water molecules. 

**Scheme 2:**
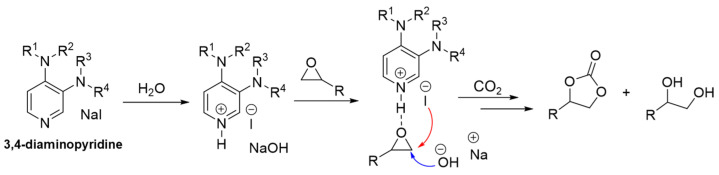
Possible effect of the addition of water on the cycloaddition reaction mechanism in the presence of strong 3,4-diaminopyridine nucleophiles. The red and blue arrows indicate that the nucleophilic attack can be carried out by the iodide or the hydroxide anions.

Natongchai et al. [55] investigated the effect of the presence of strong pyridine nucleophiles [123,124,125] on the cycloaddition of CO_2_ to epoxides catalyzed by group I and II metal halides under mild conditions (60 °C, 1 bar). While the latter halides are generally sluggish catalysts for the target process, the presence of strong nucleophilic organocatalysts such as DMAP (*N,N*-Dimethyl-4-aminopyridine) or of other variously substituted 3,4-diaminopyridine scaffolds boosted the catalytic performance of readily available salts such as MgI_2_ and even sodium-based NaI, leading to high carbonate yields and selectivity under atmospheric pressure (Table 1, entries 9, 10). Given the beneficial effect of the presence of water on the catalytic performance of halide salts [113,114], as discussed above, the authors investigated the effect of the addition of several equivalents (1–10) of water relative to the metal halides. Firstly, the authors showed that, under mild reaction conditions, the use of NaI (1 mol%) in the presence of as much as 10 mol% water did not have any catalytic activity in the cycloaddition reaction of CO_2_ to styrene oxide (Table 1, entry 11). On the other hand, when one to ten equivalents of water were added to the reaction catalyzed by NaI and a 3,4-diaminopyridine analogue of DMAP (Table 1, entry 12), the substrate conversion slightly increased to about 80%, and the carbonate selectivity slightly dropped, independent of the amount of water added. Therefore, in the presence of strong nucleophiles, the catalytic enhancement obtained by the addition of water was very limited and partially compromised the reaction selectivity. In the presence of strong nucleophiles such as aminopyridines, the addition of water is likely to lead to the protonation of the nucleophile and formation of pyridinium hydroxide (Scheme 2). 

While the pyridinium cation can serve as an HBD, and the formed hydroxide as an additional nucleophile [57], the presence of a strong alkali base (NaOH) favors the epoxide hydrolysis and cyclic carbonate decarboxylation [39,126]. These data clearly show that the effect of water on the catalytic reaction is strongly influenced by the reaction conditions and by the nature and the chemical properties of the other catalytic components. 

Imidazolium halide salts are active bifunctional catalysts for the cycloaddition of CO_2_ to epoxides because of the simultaneous presence of acidic C2 protons acting as HBDs and halide nucleophiles [127]. Their catalytic performance is influenced by the occurrence of ion-pairing between the halide ions and the positively charged imidazolium ring [117] which can be reduced by increasing the steric hindrance of the ring through the functionalization with bulky alkyl substituents [128]. Bobbink et al. reported a detailed exploration of the mechanistic aspects of the cycloaddition process catalyzed by 1-butyl-3-methylimidazolium halide salts (ImX, X = halide anion) [117]. The authors found that the addition of small amounts of water (5 mol%) to the reaction had limited beneficial effects on the cycloaddition of CO_2_ epichlorohydrin catalyzed by ImX with X = Br, I, while it was detrimental for X = Cl due to the different solvation effects of the halide ions by water as discussed above. Importantly, the improvement in catalytic performance for ImI in the presence of water was far less profound than for TBAI (Table 1, entries 13–16), for which the addition of water led to a substrate conversion even slightly higher than for ImI, due to the competition between water and the slightly acidic protons of the imidazole ring as HBDs. Valverde et al. investigated differently substituted resin-supported imidazolium chloride salts for the cycloaddition of CO_2_ to styrene oxide [129]. While the authors did not directly study the effect of the addition of water, they investigated the water content of the resins by thermogravimetry. They found a strong correlation between the water content of the resin catalysts and catalytic performance of the different immobilized imidazolium compounds, showing that a small variations in the water content (between 0.3 and 0.7 mol% relative to the epoxide) accounted for remarkable improvements in catalytic performance (up to a 40–50% increase in epoxide conversion). According to such a study, the difference in water content of the resins had a much stronger effect than the structural differences between the imidazolium compounds.

**Scheme 3:**
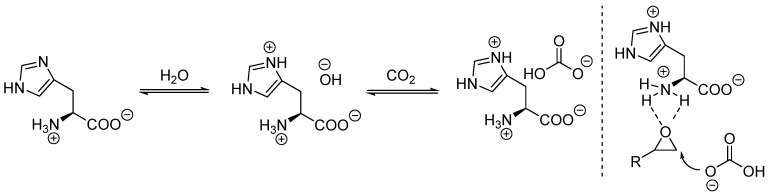
Proposed equilibria of bicarbonate anion formation in the cycloaddition of CO_2_ to epoxides by His/water (**left**) and mechanism of epoxide activation by His and bicarbonate (**right**).

The synthesis of cyclic carbonates by halide-free organocatalysts is regarded as a more environmentally friendly approach than the traditional use of organohalides due to the toxicity of halides and the carbon footprint associated with their production [48,49,130,131]. Moreover, the use of biobased organocatalysts in place of their synthetic counterparts is regarded as sustainable and cost-competitive [46,47]. Therefore, Tharun et al. revisited the cycloaddition of CO_2_ to epoxides by simple natural amino acids [118] by applying milder reaction conditions than in previous studies [132]. Interestingly, while at 120 °C, 12 bar CO_2_ no propylene oxide conversion to propylene carbonate was observed for any amino acid, the addition of water (~13 mol%) led to the formation of the target product. In particular, high propylene oxide conversion and propylene carbonate selectivity were observed for basic amino acid histidine (His, Table 1, entries 17, 18). In general, basic amino acids such as histidine, arginine, and lysine were by far more active than acidic amino acids such as aspartic and glutamic acid. The observed catalytic activity in the presence of water was attributed to its potential role as an HBD. However, the structure of His already contains a more acidic, and, therefore, stronger [133], HBD than water; i.e., the carboxylic acid group (or the protonated ammonium group) with a p*K*_a_ of about 7 expected to be more active than water in promoting the cycloaddition process [14,134]. Instead, amino acids lack a strong nucleophilic moiety for the ring-opening step of the epoxide. Therefore, the main role of water in the His-catalyzed cycloaddition is likely to provide hydroxide anions upon deprotonation by the basic imidazole groups that are subsequently transformed into nucleophilic bicarbonate anions by a reaction with CO_2_ according to the mechanism reported for quaternary ammonium hydroxides (Scheme 3) [57].

Overall, the data shown in this section and Table 1 demonstrate that, for catalytic systems that are completely devoid of H-bonding moieties such as simple organic and inorganic halide salts, the addition of water is crucial to enhance the catalytic performance because it serves as an HBD. For instance, the rate of the cycloaddition reaction of CO_2_ to propylene oxide by PPh_3_BuI was accelerated by a factor of seven in the presence of water; similarly, the cycloaddition of CO_2_ to epoxides catalyzed by NaI or histidine did not take place even under harsh conditions but was successful, under the same conditions, in the presence of water (Table 1, entries 5, 6 and 17, 18). However, it is worth noting that, when other, more efficient, HBD groups are available, such as in the case of ImI, the effect of the addition of water is less remarkable. Moreover, the actual role and the ultimate effect of the presence of water depends on the kind of halide anion (with iodide displaying the strongest improvement in performance due to the poor solvation by water) and on the chemical properties of the other catalytic components.

### 2.3. Cycloaddition of CO_2_ to Epoxides in Aqueous Biphasic Systems

In this section, the progressive development, in recent years, of the biphasic cycloaddition of CO_2_ to epoxides as a way to facilitate catalyst separation and/or recycling and to accelerate the catalytic process will be discussed. To note, this section will not include cases where water was not used as a reaction component but was added at the end of the reaction for the purpose of product purification [116], or for extracting the catalyst [135].

**Scheme 4:**
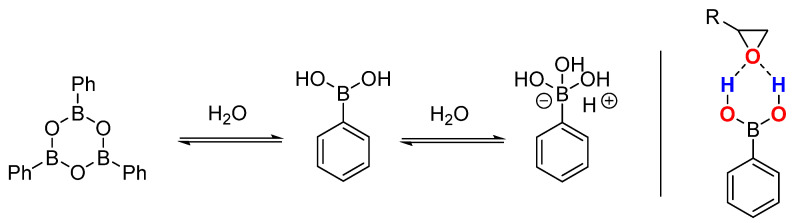
Equilibria between oligomeric and monomeric phenyl boronic acids in water (**left**) and epoxide activation by phenyl boronic acid (**right**).

In an early work, Wang and Zhang reported the cycloaddition of CO_2_ to epoxides catalyzed by a variety of phenyl boronic acids (10 mol%) as HBDs with TBAI as the source of nucleophilic halide anions (5 mol%) [136]. Because boronic acids tend to form cyclic-anhydride-type oligomers that would inhibit their role as HBDs, the authors carried out the cycloaddition process in a large excess of water (2 mL for 2 mmol substrate) as a “solvent” at 50 °C, 10 bar CO_2_ pressure to shift the equilibrium towards the hydrated form of the boronic acids (Scheme 4). Accordingly, a nearly 90% glycidyl phenyl ether conversion was observed under aqueous conditions versus not over 60% observed with any organic solvent. Under the applied aqueous conditions, given the lack of solubility of most epoxides in water, the reactions likely took place, at least initially, in a dispersion of epoxide droplets in water. Indeed, the authors claimed that the carbonate products were “automatically” separated from the aqueous phase at the end of the reaction. To the best of our knowledge, no attempts were carried out in recycling the molecular boronic acids or the aqueous layer containing the boronic acids.

“On water” processes are recognized as efficient methodologies to accelerate chemical reactions by carrying them out in suspensions of tiny droplets of concentrated organic materials surrounded by water [87,90]. In the literature, the acceleration of the chemical reactions is attributed to hydrophobic effects constraining the organic reagents in a confined space leading to the formation of more compact transition states [90,105], and to the organization of the reagents via H-bond by water [88,92], and other added HBD catalysts [93]. Despite these advances, to the best of our knowledge, the cycloaddition of CO_2_ to epoxides through an “on water” approach had not been carried out until a recent work by Jaroonwatana et al. [137]. The authors proposed that an “on water” process could serve to accelerate the carbonation of hydrophobic long-chain terminal epoxides whose cycloaddition products find application as non-ionic surfactants [36]. Importantly, the cycloaddition of CO_2_ to long-chain terminal epoxides (specifically 1,2-epoxidodecane) could not be efficiently carried out under atmospheric pressure by using TBAI as the catalyst at 60 °C in the absence of water or in the presence of small amounts of water as HBD according to a previous protocol developed by Alassmy et al. (Table 2, entries 1, 2), but originally developed under harsher reaction conditions (90 °C, 10 bar) [36]. Similarly, when the authors used a water/epoxide ratio of 8:1 (*w*/*w*) in an “on water” process with TBAI as the catalyst, only a very low substrate conversion was observed with low carbonate selectivity indicating that the reaction took place in the aqueous layer leading to epoxide ring-opening (Table 2, entry 3). In order to stabilize the organic dispersion formed after sonication in the aqueous medium, the authors tested the addition of surfactants such as sodium dodecyl sulfate (SDS) and *N,N*-dimethyldodecylamine (DDA). The results of the addition of such surfactants (Table 2, entries 4, 5) show that, while no improvement in catalytic performance was obtained with SDS, the presence of DDA in the “on water” process allowed the reaction to proceed under atmospheric pressure in high yields and complete selectivity for the carbonate product. The importance of DDA as a long-chain amine surfactant was demonstrated by replacing it with triethylamine which did not allow the efficient conversion of the substrate under “on water” conditions (Table 2, entry 6). The developed catalytic protocol could be extended to a series of long-chain epoxides with different side chain lengths. The emulsion was easily resolved at the end by the addition of dichloromethane leading to two phases (organic and aqueous) that were readily separated. However, no attempt was carried out to reuse the aqueous phase that contained a large part of the TBAI catalyst and part of the surfactant.

**Figure 2:**
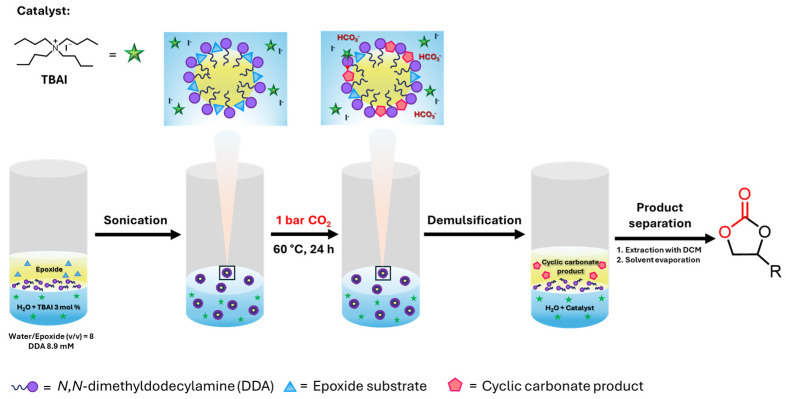
Mechanistic depiction of the “on water” cycloaddition of CO_2_ to long-chain epoxides catalyzed by TBAI in the presence of DDA as a surfactant which is proposed to increase the local concentration of iodide and bicarbonate anions at the water–substrate interface.

Mechanistically, the DDA surfactant, in its protonated form in water (DDAH^+^), is expected to be located at the water–epoxide interface with the protonated ammonium group oriented towards the aqueous phase and it serves to stabilize the organic phase droplet (Figure 2). The interaction of the protonated DDA with CO_2_ is known to lead to the formation of bicarbonate anions as counterions of DDAH^+^ [138,139]. The formation of bicarbonate anions, with the latter being in equilibrium with free CO_2_, may facilitate the transport of CO_2_ to the interface for the interaction with the substrate during the interfacial TBAI-catalyzed process, allowing the reaction to take place at ambient pressure. Moreover, the anion exchange between the iodide of TBAI and the bicarbonate of DDAH^+^ may lead to an increase in the local concentration of catalytically active nucleophilic iodide anions at the interface. Overall, this work showed that the cycloaddition of CO_2_ to challenging epoxide substrates can be accelerated through biphasic aqueous processes; however, no recycling of the catalyst and the surfactants was carried out. 

**Scheme 5:**
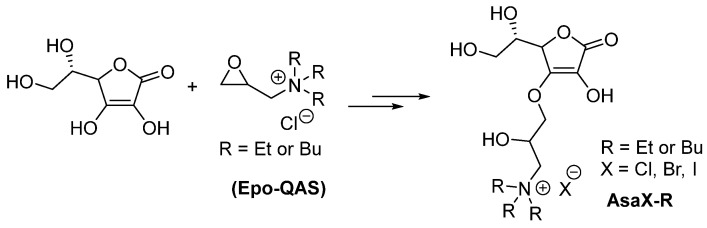
Structures of water-soluble AsaX-R HBD catalysts synthesized from ascorbic acid and quaternary ammonium halide bearing epoxy moieties (Epo-QAS).

**Figure 3:**
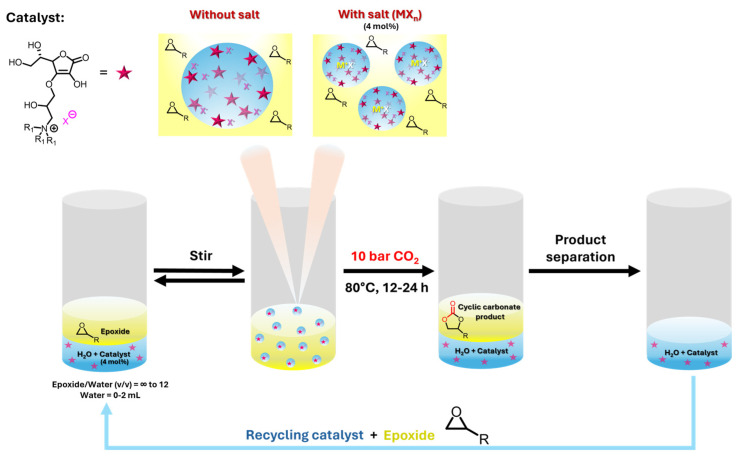
Mechanistic depiction of the “around water” cycloaddition of CO_2_ to epoxides catalyzed by AsaX-R catalysts at the interface between dispersed water droplets in the epoxide substrate. The cyclic carbonate product and the catalyst-containing aqueous phase are simply separated by decantation at the end of the process with the latter being recycled for a new catalytic run. The insets on top show the proposed role of salting-out salt additives in enhancing the reaction rate by decreasing the size of the aqueous phase droplets.

In a recent work, Theerathanagorn et al. reported the development of bifunctional water-soluble HBD ionic organocatalysts for the cycloaddition reaction of CO_2_ with epoxides in biphasic reaction systems (Scheme 5) [71]. Differently from the biphasic process discussed above, the aqueous layer formed the dispersed phase. Moreover, the catalyst was soluble exclusively in water and operated at the interface between the dispersed water droplets and the epoxide in a quasi-heterogeneous fashion. Accordingly, the authors dubbed this process as “around water” as opposed to the classical “on water” processes in which the organic layer generally represents the dispersed phase in order to induce hydrophobic interactions between the reagents. The developed HBD catalysts (Scheme 5, herein denoted as AsaX-R, X = Cl, Br, I; R = Et, Bu) were based on the versatile ascorbic acid scaffold and on its reaction with quaternary ammonium salts bearing epoxy moieties (Epo-QAS) that underwent ring-opening [63]. The AsaX-R compounds were poorly soluble in the epoxide substrate at r.t. They displayed a slightly higher solubility in the carbonate phase compared to the less polar epoxide phase, but their solubility in the carbonate products strongly decreased in the presence of an aqueous layer due to repartition. Additionally, the length of the carbon chain of the initial Epo-QAS had a relevant effect on the partial solubility of the catalysts in the organic phase. Their high solubility in water allowed their direct and atom-economic recovery and recycling in the form of aqueous solutions after the reaction without the need for solvent extractions and/or the energy-intensive evaporation of aqueous or carbonate layers that are typically required for recycling molecular catalysts such as ionic liquids and other ionic compounds (Figure 3) [140,141,142].

Although AsaI-Bu was more efficient than AsaI-Et in an initial screening carried out at 80 °C, 10 bar in the absence of added water (likely due to its higher solubility in the substrate), the lack of solubility of the latter compound in the polar reaction product made it a more suitable candidate for the application as a component of recyclable biphasic systems.

AsaI-Et was applied for the coupling reaction between CO_2_ and 1-hexene oxide in biphasic reaction systems in the presence of increasing volumes of water (Table 3, entries 1–6). Compared to the reaction carried out without adding water, the catalytic performance of AsaI-Et strongly increased just by the addition of small amounts of water due to dissolution of the catalyst (Table 3, entries 1, 2). Increasing the volume of added water led to a complete substrate conversion with complete selectivity for the carbonate product with the catalyst operating at the water/epoxide interface (Table 3, entries 3, 4). However, a further increase in the amount of water to a level nearly matching the volume of the epoxide led to a drop in catalytic performance; the catalyst was fully inactive when using an excess of water (Table 3, entries 5, 6). Such an observation was attributed to the phase inversion between water and epoxide for low volumetric epoxide/water ratios with the catalyst being sequestrated in the bulk aqueous layer. Importantly, the presence of some inorganic salts was found to increase the catalytic performance of AsaI-Et (Table 3, entries 7–12). In particular, salting-out salts [143,144] such as KI, KOAc, and NaCl strongly improved the catalytic performance of AsaI-Et independent of the nucleophilicity of the salt anion, while salting-in salts such as LiCl, NaI, and, especially, LiClO_4_ inhibited the reaction. The effect of the salting-out salts was not fully clarified but was likely due to the formation of smaller water droplets increasing the surface of the water–epoxide interface, or to a different distribution of the catalyst at the interface between the water and epoxides (See inset of Figure 3) [36,145]. Based on the observed salting-out effects, the aqueous layer was directly replaced by seawater leading to the quantitative conversion of the epoxide substrate with a high carbonate selectivity without affecting recyclability (Table 3, entry 14).

The biphasic reaction protocol could be applied to a variety of terminal epoxides generally leading to the formation of the desired carbonate products as pure phases that could be recovered by a simple decantation from the aqueous layer that was reused for further catalytic cycles.

Under harsher reaction conditions than for terminal epoxides, the biphasic reaction system could also be applied for the production of carbonated fatty acid esters (CFAEs) from the parent internal epoxides produced from the epoxidation of biobased unsaturated fatty acid esters. CFAEs serve as essential building blocks in the synthesis of various materials such as increasingly important polyhydroxy urethanes [30,32,33,146,147]. However, the development of efficient recyclable catalytic systems for their synthesis has generally proven challenging [148,149,150]. The authors showed that the developed biphasic HBD catalysts using seawater as the aqueous layer could be recycled up to five times while maintaining a high carbonated selectivity without a significant loss of activity.

## 3. Outlook and Conclusions

In the past, water was generally considered as an undesirable component of catalytic chemical reactions leading to catalyst degradation and to the formation of unwanted hydrolysis by-products [90]. With the advent of organocatalysis [101], characterized by the use of less moisture-sensitive metal-free catalysts, a strong trend towards the development of reaction protocols carried out in water or in the presence of water has emerged [87].

Despite the successful use of water as a reaction component or medium in many processes, reports on the crucial nonreductive cycloaddition of CO_2_ to epoxides carried out in aqueous systems or even in the presence of added water have thus far remained sparse. However, we have shown, in this work, that water can play a crucial role in enhancing the catalytic cycloaddition reaction, although its role and overall effect on the process strongly depend on the nature of the catalyst and on its chemical properties. In cycloaddition reactions catalyzed by ionic systems such as organic and inorganic halide salts, which exclusively bear nucleophilic halides as active catalytic moieties, water performs as a hydrogen bond donor providing the activation of the epoxide that is crucial for the acceleration of the process. For this kind of aqueous system, the main challenge is to develop more efficient catalytic protocols by using water in partnership with ionic compounds or ionic liquids with a higher catalytic performance than traditionally employed quaternary ammonium halide salts such as TBAI. The use of such ionic compounds and the suitable amount of water as an additive should lead to a high selectivity for cyclic carbonates versus diols by employing mild reaction conditions (T = 60–80 °C).

In future endeavors, one attractive way to use water in the target cycloaddition process could be to enhance the activity of recyclable heterogeneous systems bearing halide-based nucleophilic ionic groups, while negating the need for more sophisticated single-component systems. Indeed, while ionic polymers for the cycloaddition of CO_2_ to epoxides are generally easily accessed [10,151,152,153], the construction of co-immobilized catalytic systems bearing both nucleophilic halides and HBD moieties is generally way more challenging in terms of the costs and required synthetic steps [154,155]. Although water is not as active as a HBD compared to more acidic compounds such as phenols and catechols, that allow the cycloaddition process to proceed under very mild conditions [14,155,156], it can be used to boost the catalytic performance of simple ionic polymers, resulting in viable inexpensive catalysts. In this line of research, Xu et al. have recently shown that readily accessible ionic polymers able to absorb relatively large amounts of moisture (13.2 wt%) have the ability to carry out the coupling of CO_2_ and epoxides under mild and even ambient conditions due to the presence of water as an HBD in the reaction [153]. Therefore, it is recommended that water be regularly added as an HBD when exploring the activity of heterogeneous ionic organocatalysts for the target reaction as a way to boost their activity.

However, the role of water in the cycloaddition of CO_2_ to epoxides goes beyond that of an HBD as it can be used in “on water” processes to accelerate the carbonation of terminal epoxides or in recently developed “around water” processes to form a dispersed and recyclable catalyst-containing aqueous phase. For the case of the cycloaddition of CO_2_ to epoxides in a biphasic system, future efforts will surely be focused on enhancing the efficiency and scope of both “on water” and “around water” processes. For the former protocol, despite the seminal report described above, many aspects remain unexplored. Given the importance of the amine surfactant in promoting the reaction by increasing the halide and/or CO_2_ concentration at the interface, it would be crucial that we combine the organic halide catalyst and the amine surfactant into a single molecule or solid support to avoid the need for two distinct components. Such a step is also crucial for the development of recoverable systems for the “on water” cycloaddition reaction, perhaps, by generating compounds that are heterogeneous or soluble exclusively in the aqueous layer and are recycled directly in the form of aqueous solutions. Moreover, more potent catalysts should be developed for the “on water” process to allow the carbonation of other classes of hydrophobic but unreactive internal epoxides such as epoxidized fatty acids and vegetable oil that are becoming increasingly sought after in sustainable polymer chemistry.

Similarly, despite the first successful implementation of the “on water” process, several aspects remain unexplored. Thus far, only a single family of ascorbic-acid-derived hydrophilic catalysts was utilized, whereas other polar, water-soluble scaffolds such as sugars and amino acids could be modified to work at the water–substrate interface. Another crucial aspect is to clarify the role of salt additives and their interaction with the catalyst. Given the strong boost of catalytic activity observed in the presence of salting-out additives, it is worth investigating how the droplets’ size and the catalyst distribution in the droplets are influenced by the presence of ionic and nonionic additives, for instance, through fluorescence microscopy [157]. Additives that are able to increase the local concentration of catalyst at the interface without “salting it out” to the organic phase are likely to induce a strong boost in catalytic performance.

Overall, water is potentially a powerful ally in the organocatalytic cycloaddition of CO_2_ to epoxides to enhance the catalytic activity through hydrogen-bonding and/or hydrophobic interactions or to facilitate the “in flask” recovery of molecular catalysts. Therefore, we anticipate and recommend its increasing involvement in the target process. 

## Figures and Tables

**Table 1:**
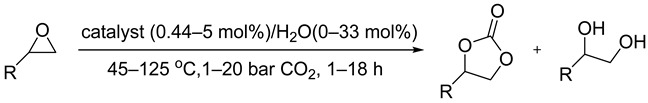
Overview of results of cycloaddition reactions carried out in the presence of substoichiometric amounts of water as an additive.

Entry	R	Catalyst (mol%)	H_2_O (mol%)	T, P (°C), (bar)	Time (h)	Yield ^1^ (%)	Select. (%)	Ref.
1	-CH_3_	TBAI (0.5)	33	125, 20	1	95	88	[113]
2	-CH_3_	TBAI (0.5)	-	125, 20	1	27	26	[113]
3	-CH_3_	PPh_3_BuI (0.5)	33	125, 20	1	100	95	[113]
4	-CH_3_	PPh_3_BuI (0.5)	-	125, 20	1	25	24	[113]
5	-CH_3_	NaI (1)	-	120, 20	1	8	>99	[114]
6	-CH_3_	NaI·2H_2_O (1)	-	120, 20	1	94	>99	[114]
7	-CH_3_	TBAI (1)	14	45, 10	18	55	>99	[116]
8	-CH_3_	PPNI (1)	14	45, 10	18	59	>99	[116]
9	-Ph	MgI_2_ (1)/ Aminopyridine ^2^ (2)	-	60, 1	12	77	99	[55]
10	-Ph	NaI (1)/ aminopyridine (2)	-	60, 1	12	61	93	[55]
11	-Ph	NaI (1)	10	60, 1	12	-	-	[55]
12	-Ph	NaI (1)/ aminopyridine (2)	1–10	60, 1	12	~80	88	[55]
13	-CH_2_Cl	ImI ^3^ (5)	-	50, 1	3	62	-	[117]
14	-CH_2_Cl	ImI (5)	5	50, 1	3	65	-	[117]
15	-CH_2_Cl	TBAI (5)	-	50, 1	3	36	-	[117]
16	-CH_2_Cl	TBAI (5)	5	50, 1	3	68	-	[117]
17	-CH_3_	His ^4^ (0.44)	-	120, 12	3	-	-	[118]
18	-CH_3_	His (0.44)	13	120, 12	3	90	91	[118]

^1^ Yield or conversion depending on the original source. ^2^ 3,4-diaminopyridine analogue of DMAP. ^3^ ImI: 1-butyl-3-methylimidazolium iodide. ^4^ Histidine.

**Table 2:**
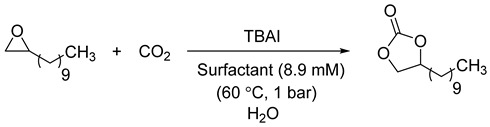
Overview of “on water” cycloaddition of CO_2_ to 1,2-epoxidodecane and related control experiments as reported by Jaroonwatana et al. [137].

Entry	Water/Epoxide (*v*/*v*)	Surfactant	Conversion (%)	Selectivity (%)
1	-	-	3	100
2	0.005	-	5	100
3	8	-	3	45
4	8	SDS	2	50
5	8	DDA	87	≥99
6	8	TEA	3	100

**Table 3:**
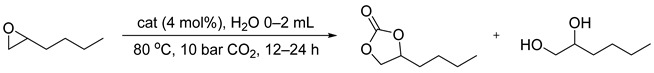
The effect of amount of water and salt additives on the coupling reaction between CO_2_ and 1-hexene oxide catalyzed by HBD AsaI-Et (cat) in biphasic media.

Entry	H_2_O (mL)	Epoxide/Water (*v*/*v*)	Salt Additive *^a^*	Time (h)	Conversion (%)	Selectivity (%)
1	-	∞	-	24	15	88
2	0.1	12	-	24	83	99
3	0.5	2.4	-	24	99	99
4	0.8	1.5	-	24	99	98
5	1.0	1.2	-	24	89	96
6	2.0	0.6	-	24	4	41
7	0.5	2.4	-	12	35	94
8	0.5	2.4	KI	12	87	98
9	0.5	2.4	KOAc	12	99	97
10	0.5	2.4	NaCl	12	77	97
11	0.5	2.4	LiCl	12	30	91
12	0.5	2.4	NaI	12	28	74
13	0.5	2.4	LiClO_4_	12	7	26
14 *^b^*	-	2.4	Seawater	12	99	99

*^a^* Salt (4 mol%) was added to the aqueous layer. *^b^* Using 0.5 mL seawater instead of DI water.

## Data Availability

No new experimental data were created for this work.
